# Artificial neural networks modeling gene-environment interaction

**DOI:** 10.1186/1471-2156-13-37

**Published:** 2012-05-14

**Authors:** Frauke Günther, Iris Pigeot, Karin Bammann

**Affiliations:** 1 BIPS - Institute for Epidemiology and Prevention Research GmbH, Bremen 28359, Achterstraße 30, Germany; 2 University of Bremen, Institute of Public Health and Nursing Science (IPP), Bremen 28359, Grazer Straße 4, Germany

**Keywords:** Gene-environment interaction, Multilayer perceptron, MLP, Neural network, Pattern recognition, Simulation study

## Abstract

**Background:**

Gene-environment interactions play an important role in the etiological pathway of complex diseases. An appropriate statistical method for handling a wide variety of complex situations involving interactions between variables is still lacking, especially when continuous variables are involved. The aim of this paper is to explore the ability of neural networks to model different structures of gene-environment interactions. A simulation study is set up to compare neural networks with standard logistic regression models. Eight different structures of gene-environment interactions are investigated. These structures are characterized by penetrance functions that are based on sigmoid functions or on combinations of linear and non-linear effects of a continuous environmental factor and a genetic factor with main effect or with a masking effect only.

**Results:**

In our simulation study, neural networks are more successful in modeling gene-environment interactions than logistic regression models. This outperfomance is especially pronounced when modeling sigmoid penetrance functions, when distinguishing between linear and nonlinear components, and when modeling masking effects of the genetic factor.

**Conclusion:**

Our study shows that neural networks are a promising approach for analyzing gene-environment interactions. Especially, if no prior knowledge of the correct nature of the relationship between co-variables and response variable is present, neural networks provide a valuable alternative to regression methods that are limited to the analysis of linearly separable data.

## Background

The etiological pathway of any complex disease can be described as an interplay of genetic and non-genetic underlying causes (e.g. [1-3]). Usually, regression based methods are applied in the study of complex diseases (e.g. [4-8]). However, regression methods do not necessarily capture the complexity of the interplay of genetic and non-genetic factors. In particular, regression models require pre-processing of data to reflect any non-linear relationship. First, continuous variables have to be either categorized or transformed according to their assumed form of relationship to the response. Second, interaction terms have to be explicitly included into the regression models to test for any statistical interaction. Third, if no prior knowledge of the functional form of the dose-response-relationship is present, a variety of regression models has to be explored. With increasing number of variables, finding the best model through trial-and-error is no longer feasible due to the large number of possible models.

For modeling complex relationships, especially with little prior knowledge of the exact nature of these relationships, a more flexible statistical tool should be used. One promising alternative is the use of artificial neural networks. Here, variables do not have to be transformed a priori and interactions are modeled implicitly, that is, they do not have to be a priori formulated in the model [9]. We successfully applied neural networks for modeling different two-locus disease models, i.e. different types of gene-gene interactions as e.g. epistatic models [10].

Since studies using neural networks for modeling continuous co-variables have previously shown promising results (see e.g. [11-13]), the aim of this paper is to investigate the usability of neural networks for modeling complex diseases that are determined by a gene-environment interaction with a continuously measured environmental factor. Based on simulated data in a case-control design, we analyze the general modeling ability of neural networks for different structures of gene-environment interactions. Theoretic risk models are defined representing different types of two-way interactions of one genetic and one environmental factor (e.g. [14]). The predicted risk is compared to the theoretic risk to assess the modeling ability. Additionally, neural networks are trained to a real data set to investigate the practicability of neural networks in a real life situation. All results are compared to those obtained by logistic regression models as reference method. Advantages and disadvantages of using a neural network approach are discussed.

## Methods

### Simulation study

Case-control data sets are generated using a two step design. First, underlying populations are simulated with a controlled prevalence of 10% and an overall sample size of five million observations. These populations carry the information of two marginally independent and randomly drawn factors – one biallelic locus and one continuous environmental factor – and a case-control status. The minor allele frequency is 30% to ensure sufficient cell frequencies in the final case-control data sets and it is assumed that the Hardy-Weinberg equilibrium holds. The environmental factor follows a continuous uniform distribution on the interval [0,100]. Depending on the genotype *G* and the environmental factor *U*, the case-control status is allocated through eight given theoretic risk models as introduced in the next subsection. Considering each theoretic risk model in a high and a low risk scenario, this results in sixteen underlying populations. As the second step, 100 case-control data sets are randomly drawn from all underlying populations for each analysis. Thus, for each analysis, mean values over 100 data sets are considered in sixteen situations. Three different sample sizes of 2,000 subjects (1,000 cases + 1,000 controls), 1,000 subjects (500 cases + 500 controls), and 400 subjects (200 cases + 200 controls) are used.

Artificial neural networks and logistic regression models are fitted to the data, i.e. separately to all 100 case-control data sets for each situation. A multilayer perceptron (MLP, see e.g. [15]) is chosen as neural network. It is briefly described in the Appendix. For neural networks, the genotype information is coded co-dominant, i.e. the genotype takes possible values 0, 1, and 2 representing the number of mutated alleles. The environmental factor is included in the analyses as continuous variable. For all data sets, six different network topologies, from zero up to five hidden neurons, are trained to avoid an overfitting of the data. For training purposes, the data set is always used as a whole. Each training process is replicated five times each with randomly initialized starting weights drawn from a standard normal distribution to enhance the chance that the training process stops within a global instead of a local minimum. The best trained neural network for each data set, i.e. the best network topology and the best repetition, is selected based on the Bayesian Information Criterion (BIC, [16]), which takes the number of parameters into account and penalizes additional parameters. Thus in each situation, 100 best neural networks predict the underlying risk model and the mean prediction can be used to evaluate the model fit (see below).

For comparison purposes, logistic regression models are fitted to the same data sets. The genotype is coded co-dominant counting the number of risk alleles and using two dichotomous design variables, one representing the heterozygous and one representing the homozygous mutated genotype. Five different models are used: the null model, three main effect models – containing only one or both main effects – and the full model – containing both main effects and one or two interaction terms depending on the genotype coding. For both coding approaches, the best model is selected based on BIC.

To assess the model fit of neural networks and logistic regression models, the mean prediction over the 100 data set is compared to the theoretic risk model of a case-control data set. This theoretic risk model stands for a perfectly drawn case-control data set since it reflects the probabilities of the given population and takes into account the changing prevalence in a balanced case-control data set. Mean absolute differences between the theoretic risk model and its predictions are calculated element-wise for an equidistant vector (*u*^*′ *^= 0, 0.1, 0.2,…,100) used as an environmental factor which yields the matrix *E* defined as:

(1) $$E={\left(E_{gu^{\prime }}\right)}_{g,u^{\prime }}={\left(\frac{1}{100}\overset{100}{\underset{k=1}{\sum }}\left|f(g,u^{\prime })-{\hat{f}}^{\left(k\right)}(g,u^{\prime })\right|\right)}_{g,u^{\prime }},$$

where *g *= 0,1,2 denotes the genotype and *f*(*g*,*u*^*′*^) refers to the theoretic risk model of the case-control data set and ${\hat{f}}^{\left(k\right)}(g,u^{\prime })$ to the prediction of the *k*th case-control data set. The smaller $\underset{gu^{\prime }}{\sum }E_{gu^{\prime }}$ is, the better the mean model fit of neural networks or logistic regression models is since the estimated risk model and the theoretic risk model coincide for $\underset{gu^{\prime }}{\sum }E_{gu^{\prime }}=0$. To take variation into account, pointwise prediction intervals are calculated as empirical 95% intervals. In particular, for all *u*^*′ *^= 0, 0.1, 0.2,…,100 and *g *= 0,1,2 a prediction interval is determined as the interval $\left[\hat{f}{\left(g,u^{\prime }\right)}_{\left(3\right)};\;\hat{f}{\left(g,u^{\prime }\right)}_{\left(98\right)}\right]$, where $\hat{f}{\left(g,u^{\prime }\right)}_{\left(3\right)}$ and $\hat{f}{\left(g,u^{\prime }\right)}_{\left(98\right)}$ denote the 3rd ordered and the 98th ordered prediction, respectively.

Data generation and all analyses are done using R [17]. The package for training the MLP was implemented by our group and is published on CRAN [18].

### Theoretic risk models

Two different types of theoretic risk models for gene-environment interactions are used, namely the models introduced by Amato et al. [14] and models mainly representing a masking effect of the involved locus as defined below. For all risk models, the kind of functional relationship between the penetrance and the environmental factor depends on the genotype information, i.e. the curve shape is in general different depending on the three genotypes. The relationship is defined on a population level, i.e. the penetrance function *F* :{0,1,2} × [0,100] → [0,1] with *F*(*gu*) =* P*(*Y *= 1|*G *=* gU *=* u*), where *Y *∈ {0,1} denotes the case-control status, *G *∈ {0,1,2} the genotype, and *U *∈ [0,100] the environmental factor, only holds in the corresponding underlying population and has to be converted to *f*(*gu*) if a case-control data set is analyzed [10].

#### Risk models by Amato et al

Amato et al. [14] introduced four different risk models for analyzing gene-environment interactions: a genetic model, an environmental model, an additive model and an interaction model that are characterized by the following penetrance function

$$\begin{array}{l} F(g,u)=\frac{1}{1+\text{exp}\{\alpha_{g}+\beta_{g}\cdot u\}},\;\\ \;g=0,1,2;\;u\in \left[0,100\right]. \end{array}$$

The four models are defined as follows: 

• the genetic model: *α*_1_ ≤* α*_2_ ≤* α*_3_ and *β*_1_ =* β*_2_ =* β*_3_ = 0,

• the environmental model: *α*_1_ =* α*_2_ =* α*_3_ and *β*_1_ =* β*_2_ =* β*_3_ ≠ 0,

• the additive model: *α*_1_ ≤* α*_2_ ≤* α*_3_ and *β*_1_ =* β*_2_ =* β*_3_ ≠ 0,

• the interaction model: *α*_1_ =* α*_2_ =* α*_3_ and *β*_1_ ≤* β*_2_ ≤* β*_3_.

To be able to fix the prevalence *K* of disease, we introduce an upper bound *z* that is determined such that the prevalence is equal to *K *= 0.1:

$$F(g,u)=\frac{z}{1+\text{exp}\{\alpha_{g}+\beta_{g}\cdot u\}},$$

**Figure 1:**
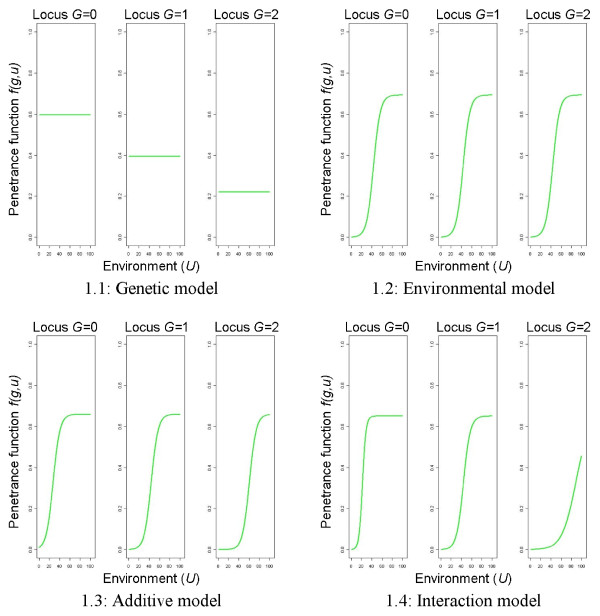
**Theoretic risk models by Amato et al. **[14]**, high risk scenario.** The left part of each figure refers to the homozygous wild-type genotype, the middle one to the heterozygous, and the right one to the homozygous mutated genotype.

The values of *α*_*g*_,*β*_*g*_, *g *= 0,1,2, and *z* used in this paper for two risk scenarios are given in Table 1. Figure 1 shows the theoretic risk models of a case-control data set *f*(*g*,*u*) for the high risk scenario.

**Table 1:** **Used values for *α***_**g**_**, *β***_**g**_**(g = 0,1,2), *c*, and z**

**Risk model**		**Risk scenario**	**Constant values *α***_**g**_, ***β***_**g**_ (** g = 0**,*1*,*2*)	**Constant values c, z**
	Genetic model	High risk	$\alpha_{0}=\frac{2}{3}\cdot \alpha_{1},\;\alpha_{1}=2.5,\;\alpha_{2}=\frac{4}{3}\cdot \alpha_{1}$	*z *= 0.886
			*β*_0_ =* β*_1_ =* β*_2_ = 0	
		Low risk	$\alpha_{0}=\frac{2}{3}\cdot \alpha_{1},\;\alpha_{1}=1.25,\;\alpha_{2}=\frac{4}{3}\cdot \alpha_{1}$	*z *= 0.390
			*β*_0_ =* β*_1_ =* β*_2_ = 0	
	Environmental model	High risk	*α*_0_ =* α*_1_ =* α*_2_ = 7.5,	*z *= 0.200
			*β*_0_ =* β*_1_ =* β*_2_ = −0.15,	
		Low risk	*α*_0_ =* α*_1_ =* α*_2_ = 3.75,	*z *= 0.200
Risk models by Amato et al. [14]			*β*_0_ =* β*_1_ =* β*_2_ = −0.075,	
	Additive model	High risk	$\alpha_{0}=\frac{2}{3}\cdot \alpha_{1},\;\alpha_{1}=7.5,\;\alpha_{2}=\frac{4}{3}\cdot \alpha_{1}$,	*z *= 0.177
			*β*_0_ =* β*_1_ =* β*_2_ = −0.15,	
		Low risk	$\alpha_{0}=\frac{2}{3}\cdot \alpha_{1},\;\alpha_{1}=3.75,\;\alpha_{2}=\frac{4}{3}\cdot \alpha_{1}$,	*z *= 0.178
			*β*_0_ =* β*_1_ =* β*_2_ = −0.075,	
	Interaction model	High risk	*α*_0_ =* α*_1_ =* α*_2_ = 7.5,	*z *= 0.171
			*β*_0_ = 2 ·* β*_1_, *β*_1_ = −0.15, *β*_2_ = 0.5·*β*_1_,	
		Low risk	*α*_0_ =* α*_1_ =* α*_2_ = 3.75,	*z *= 0.169
			*β*_0_ = 2 ·* β*_1_, *β*_1_ = −0.075, *β*_2_ = 0.5 ·* β*_1_,	
	Model 1	High risk (*r *= 0.150)		*c *= 0.05, *z *= 0.254
		Low risk (*r *= 0.075)		
Risk model representing a masking effect of the genetic factor	Model 2	High risk (*r *= 0.150)		*c *= 0.05, *z *= 0.286
		Low risk (*r *= 0.075)		
	Model 3	High risk (*r *= 0.150)		*c *= 0.075, *z *= 0.631
		Low risk (*r *= 0.075)		
	Model 4	High risk (*r *= 0.150)		*c *= 0.075, *z *= 0.964
		Low risk (*r *= 0.075)		

Constant values *α*_*g*_, *β*_*g*_(*g *= 0,1,2) *c*, and *z* used to determine the penetrance functions (minor allele frequency 30%).

#### Risk models representing a masking effect of the genetic factor

In addition, we define four theoretic risk models representing four types of gene-environment interactions where the gene mainly has a masking effect. The kind of functional relationship between the environmental factor and the penetrance again depends on the genotype information. The four theoretic risk models are described in detail in the following: 

1. The structure of the first risk model is given by the following penetrance function *F* :{0,1,2} × [0,100] → [0,1]

$$F(g,u)=\left\{\begin{array}{cc} \frac{z-c}{1+\text{exp}(-r(u-50))}+c & \text{if}g=0\\ c & \text{if}g=1.\\ c & \text{if}g=2 \end{array}\right)$$

2. The second risk model is defined by

$$F(g,u)=\left\{\begin{array}{cc} \frac{z}{1+\text{exp}(-r(u-50))} & \text{if}g=0\\ c & \text{if}g=1\;.\\ 2c & \text{if}g=2 \end{array}\right)$$

3. In the third risk model, the penetrance function is given by

$$F(g,u)=\left\{\begin{array}{cc} c & \text{if}g=0\\ c & \text{if}g=1\;.\\ \frac{z-c}{1+\text{exp}(-r(u-50))}+c & \text{if}g=2 \end{array}\right)$$

4. For the fourth risk model, the penetrance function is determined as follows:

$$F(g,u)=\left\{\begin{array}{cc} \frac{1}{2}c & \text{if}g=0\\ c & \text{if}g=1\;.\\ \frac{z-2c}{1+\exp(-r(u-50))}+2c & \text{if}g=2 \end{array}\right)$$

**Figure 2:**
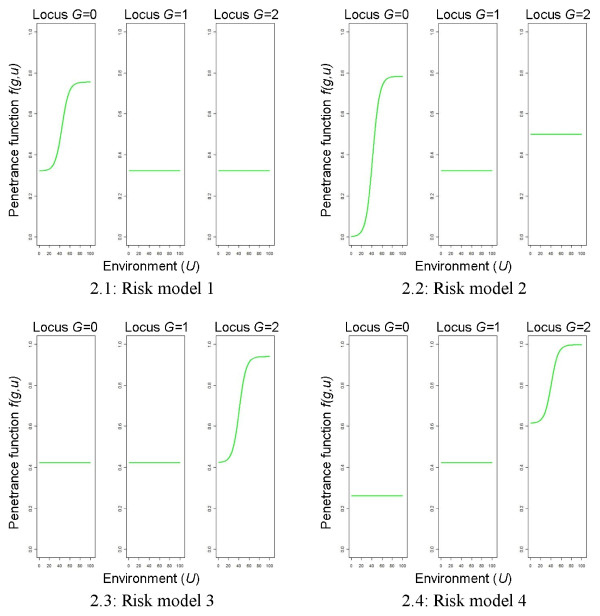
**Theoretic risk models representing a masking effect of the genetic factor, high risk scenario.** The left part of each figure refers to the homozygous wild-type genotype, the middle one to the heterozygous, and the right one to the homozygous mutated genotype.

In each of these four models, *r* denotes the risk increase, *c* a baseline risk, and *z* an upper bound for the penetrance function. A risk increase of *r *= 0.150 indicates the high risk and a risk increase of *r *= 0.075 the low risk scenario, respectively. The baseline risk *c* and the upper bound *z* are again determined such that the prevalence of disease is equal to 10% for each situation. The values used in this paper are given in Table 1. Figure 2 shows the theoretic risk models of a case-control data set *f*(*g*,*u*) for the high risk scenario. Note that the gene has a main effect on the disease in risk models 2 and 4 only and that risk model 3 differs from risk model 1 only by different cell frequencies for the three genotype classes.

### Real data application

To study the performance of a neural network in a real life situation, we applied this approach to a cross-sectional study dealing with a lifestyle induced complex disease. This application should serve as an example for the general practicability of our approach without describing the study from a subject point of view. The common effect of an SNP and a continuous environmental factor on a binary outcome is investigated while controlling for the effect of one binary confounder. The data set includes 138 cases and 1599 controls. As in the simulation study, neural networks with up to five hidden neurons are trained each five times with randomly initialized weights drawn from of a standard normal distribution and the best neural network is chosen based on BIC. The analysis is done once using the whole data set and once stratified by the confounding factor. For the stratified analysis, 95% bootstrap percentile intervals are calculated using 100 bootstrap replications [19].

## Results

### Risk models by Amato et al.

**Figure 3:**
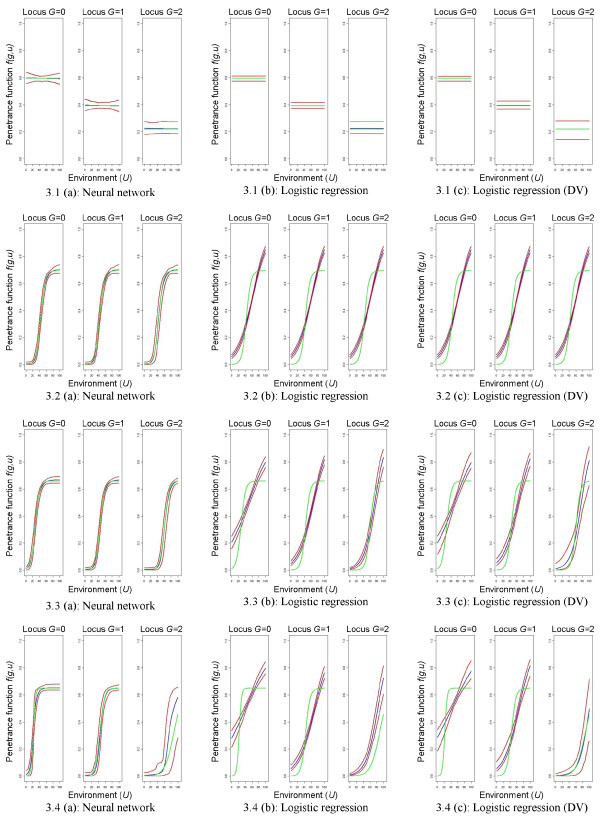
**Graphical comparison of mean predictions.** Risk models by Amato et al. [14], high risk scenario, *n *= 1,000 + 1,000. Graphical comparison of mean predictions $\frac{1}{100}\overset{100}{\underset{k=1}{\sum }}{\hat{f}}^{\left(k\right)}(g,u^{\prime })$ for all *u*^*′ *^= 0, 0.1, 0.2,…,100 and *g *= 0,1,2, where the rows relate to the different theoretic risk models. Green lines refer to the theoretic risk model, blue lines to the mean predictions, and red lines to the pointwise prediction intervals. DV = design variables.

**Figure 4:**
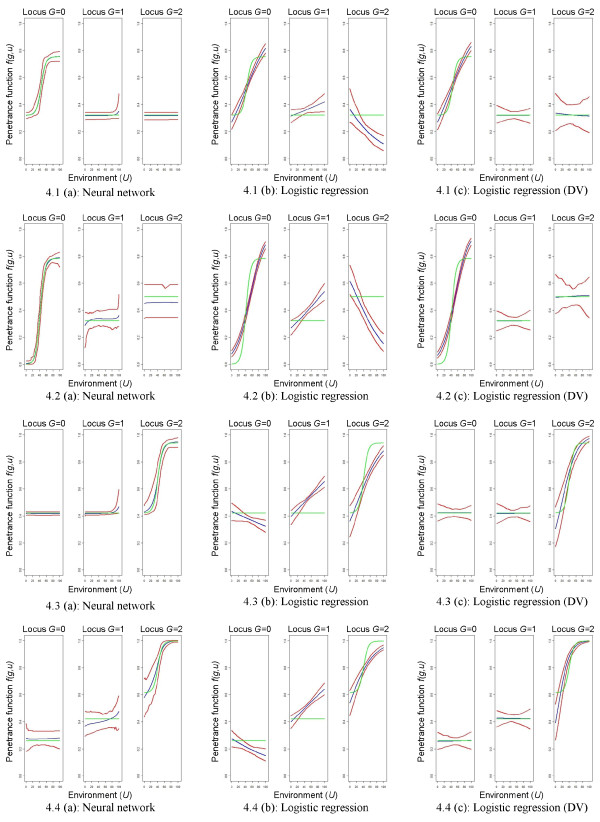
**Graphical comparison of mean predictions.** Risk models by Amato et al. [14], low risk scenario, *n *= 1,000 + 1,000. Graphical comparison of mean predictions $\frac{1}{100}\overset{100}{\underset{k=1}{\sum }}{\hat{f}}^{\left(k\right)}(g,u^{\prime })$ for all *u*^*′ *^= 0, 0.1, 0.2,…,100 and *g *= 0,1,2, where the rows relate to the different theoretic risk models. Green lines refer to the theoretic risk model, blue lines to the mean predictions, and red lines to the pointwise prediction intervals. DV = design variables.

A graphical comparison of the general modeling ability for neural networks and logistic regression models is shown in Figures 3 and 4 for the large sample size and the risk models introduced by Amato et al. [14]. In general, neural networks have a very good model fit compared to logistic regression models. Especially if the environmental factor has an effect (environmental model, additive model, interaction model), neural networks much better predict the underlying relationship between the genetic and the environmental factor with narrow prediction intervals that always include the true theoretic risk model. On the contrary, logistic regression models are only able to satisfactorily predict the genetic model. The sigmoid effects in the case that the environmental factor has an effect are not well represented in any situation and none of the prediction intervals include the theoretic risk model. This is true for both, logistic regression models with a co-dominant coding or for those using design variables for the genetic factor.

These results are also reflected by the sum of the mean absolute differences $\underset{gu^{\prime }}{\sum }E_{gu^{\prime }}$ as defined element-wise in Equation (1) (see Table 2). Bold numbers mark the best model fit comparing neural networks and logistic regression models. Neural networks have the best model fit for the environmental model, the additive model, and the interaction model in both risk scenarios and for all sample sizes. For example for the interaction model in the high risk scenario, the sum of the mean absolute differences $\underset{gu^{\prime }}{\sum }E_{gu^{\prime }}$ is less than half as large for neural networks as compared to logistic regression models ( $\underset{gu^{\prime }}{\sum }E_{gu^{\prime }}=119.77$ for neural networks as compared to $\underset{gu^{\prime }}{\sum }E_{gu^{\prime }}=345.77$ and $\underset{gu^{\prime }}{\sum }E_{gu^{\prime }}=247.93$ for logistic regression models with a co-dominant coding and with design variables). Additionally, they show the best model fit for the genetic model in the low risk scenario if the sample size is 500 + 500 or 200 + 200. Logistic regression models using a co-dominant coding show the best model fit for the genetic model in the high risk scenario and, if the sample size is 1,000 + 1,000, in the low risk scenario.

**Table 2:** **Differences between theoretic and estimated penetrance functions (models by Amato et al. [**[14]**])**

			**High risk scenario**			**Low risk scenario**	
		**Neural network**	**Logistic regression**	**Logistic regression (DV)**	**Neural network**	**Logistic regression**	**Logistic regression (DV)**
			***n =1000 + 1000***			***n=1000 + 1000***	
	Genetic model	40.79	**31.31**	48.15	48.22	**40.85**	83.62
$\underset{gu^{\prime }}{\sum }E_{gu^{\prime }}$	Environmental model	**46.14**	277.11	277.11	**52.45**	171.61	171.36
	Additive model	**45.13**	256.52	260.10	**47.99**	163.19	189.92
	Interaction model	**119.77**	345.77	247.93	**132.47**	225.61	194.37
			***n =500 + 500***			***n = 500 + 500***	
	Genetic model	59.28	**47.14**	68.22	**64.27**	92.02	159.80
$\underset{gu^{\prime }}{\sum }E_{gu^{\prime }}$	Environmental model	**60.57**	277.51	277.15	**90.76**	174.37	174.16
	Additive model	**56.10**	268.11	297.62	**80.66**	190.25	242.34
	Interaction model	**138.91**	344.50	268.75	**153.56**	233.16	210.98
			***n = 200 + 200***			***n = 200 + 200***	
	Genetic model	101.95	**85.67**	152.25	**97.23**	167.48	207.66
$\underset{gu^{\prime }}{\sum }E_{gu^{\prime }}$	Environmental model	**96.32**	278.40	278.93	**163.16**	177.14	175.27
	Additive model	**96.16**	329.55	374.17	**177.24**	246.06	292.39
	Interaction model	**168.90**	349.88	316.01	**207.81**	256.22	291.88

Sum of mean absolute differences between theoretic and estimated penetrance function for 100 case-control data sets in the low and high risk scenario for different sample sizes. Bold numbers mark the best model fit comparing neural networks and logistic regression models. DV = design variables.

If the sample size decreases, the modeling ability becomes worse for neural networks as well as for logistic regression models (see Table 2). However, neural networks still show the best model fit if the environmental factor has an effect. The prediction intervals include the true underlying risk model in all but two situations (interaction model, *n *= 500 + 500, low risk scenario and interaction model, *n *= 200 + 200, high risk scenario, data not shown).

### Models representing a masking effect of the genetic factor

**Figure 5:**
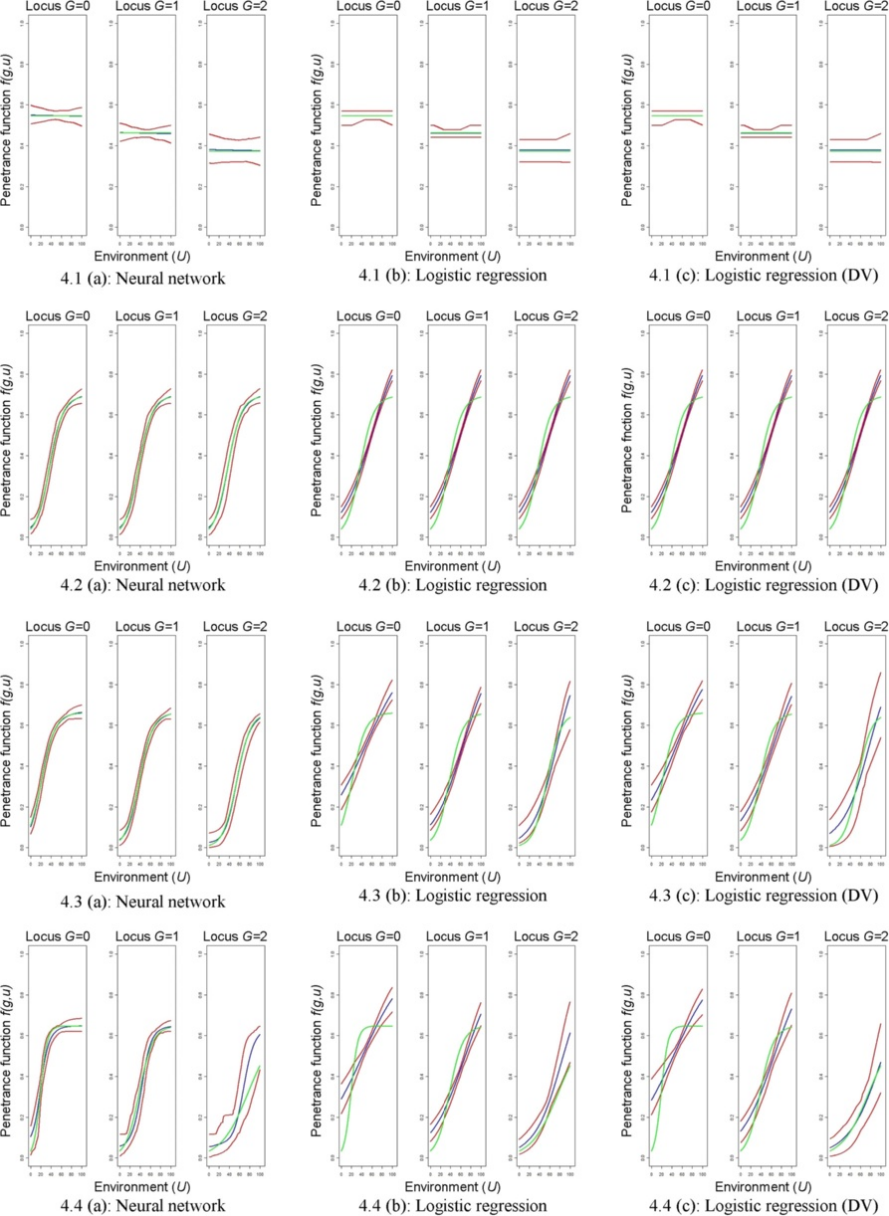
**Graphical comparison of mean predictions.** Risk models representing a masking effect of the genetic factor, high risk scenario, *n *= 1,000 + 1,000. Graphical comparison of mean predictions $\frac{1}{100}\overset{100}{\underset{k=1}{\sum }}{\hat{f}}^{\left(k\right)}(g,u^{\prime })$ for all *u*^*′ *^= 0, 0.1, 0.2,…,100 and *g *= 0,1,2, where the rows relate to the different theoretic risk models. Green lines refer to the theoretic risk model, blue lines to the mean predictions, and red lines to the pointwise prediction intervals. DV = design variables.

**Figure 6:**
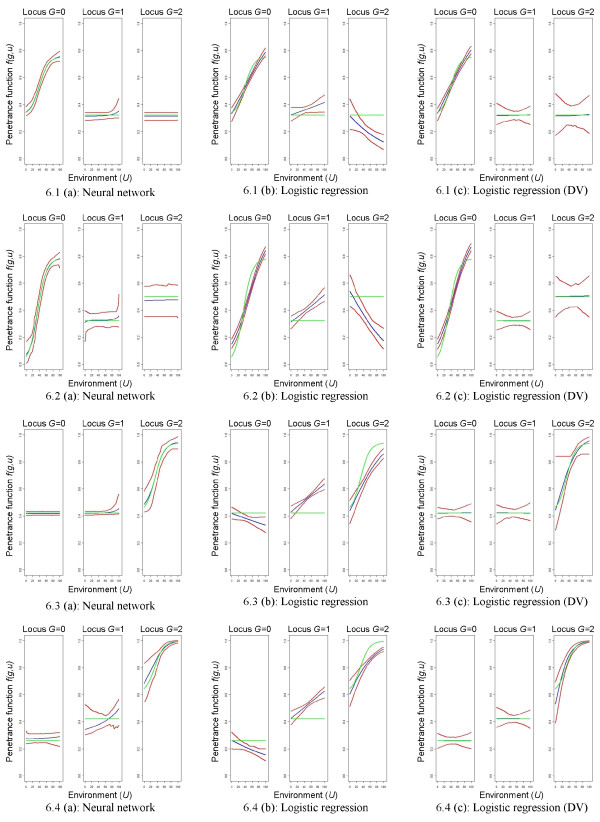
**Graphical comparison of mean predictions.** Risk models representing a masking effect of the genetic factor, low risk scenario, *n *= 1,000 + 1,000 Graphical comparison of mean predictions $\frac{1}{100}\overset{100}{\underset{k=1}{\sum }}{\hat{f}}^{\left(k\right)}(g,u^{\prime })$ for all *u*^*′ *^= 0, 0.1, 0.2,…,100 and *g *= 0,1,2, where the rows relate to the different theoretic risk models. Green lines refer to the theoretic risk model, blue lines to the mean predictions, and red lines to the pointwise prediction intervals. DV = design variables.

The general modeling ability for the risk models representing a masking effect of the genetic factor is shown in Figures 5 and 6 for the large sample size. Neural networks have a very good model fit if the gene has a masking effect only (risk model 1 and 3). If the gene has an own main effect, the prediction is less accurate and the variance is much larger. Nevertheless, the prediction intervals include the true theoretic risk models in all situations. Logistic regression models with a co-dominant coding for the genotype are not able to capture the underlying structure of the gene-environment interaction. Constant or non-linear effects are not detected. If design variables are used for coding the genotype, the model fit is much better. However, the theoretic risk model is not included in the prediction interval in many situations. Additionally, the constant effects are only detected by combining the single predictions with either positive or negative slopes to an average prediction with zero slope. This is reflected by the wider ends of the prediction intervals. Moreover, the sigmoid effect is again not well represented in many of the investigated situations. This is especially true for the first and the second risk model.

Comparing the sum of the mean absolute differences $\underset{gu^{\prime }}{\sum }E_{gu^{\prime }}$ (see Table 3), neural networks show the best model fit to the underlying data for the first three risk models if the sample size is *n*=1,000 + 1,000, thus, representing best the gene-environment interactions in these situations. For example for risk model 1 in the high risk scenario, the sum of the mean absolute differences is $\underset{gu^{\prime }}{\sum }E_{gu^{\prime }}=38.63$ for neural networks as opposed to $\underset{gu^{\prime }}{\sum }E_{gu^{\prime }}=211.62$ and $\underset{gu^{\prime }}{\sum }E_{gu^{\prime }}=105.83$ for logistic regression models with a co-dominant coding and with design variables. For risk model 4, logistic regression models using design variables for the genotype clearly have the best model fit ( $\underset{gu^{\prime }}{\sum }E_{gu^{\prime }}=85.16$ for the high and $\underset{gu^{\prime }}{\sum }E_{gu^{\prime }}=59.74$ for the low risk scenario as opposed to $\underset{gu^{\prime }}{\sum }E_{gu^{\prime }}=103.37$ and $\underset{gu^{\prime }}{\sum }E_{gu^{\prime }}=103.63$ for neural networks).

**Table 3:** Differences between theoretic and estimated penetrance functions (models representing a masking effect of the genetic factor)

			**High risk scenario**			**Low risk scenario**	
		**Neural network**	**Logistic regression**	**Logistic regression (DV)**	**Neural network**	**Logistic regression**	**Logistic regression (DV)**
			***n = 1000 + 1000***			***n = 1000 + 1000***	
	Model 1	**38.63**	211.62	105.83	**41.07**	195.15	87.57
$\underset{gu^{\prime }}{\sum }E_{gu^{\prime }}$	Model 2	**117.94**	359.10	155.40	**101.92**	323.89	114.71
	Model 3	**40.67**	253.01	85.51	**43.15**	258.19	65.87
	Model 4	103.37	228.10	**85.16**	103.63	227.50	**59.74**
			***n = 500 + 500***			***n = 500 + 500***	
	Model 1	**54.58**	219.39	136.26	**70.40**	207.97	140.74
$\underset{gu^{\prime }}{\sum }E_{gu^{\prime }}$	Model 2	**144.35**	363.36	176.74	183.28	327.58	**143.06**
	Model 3	**60.98**	261.86	110.93	**66.25**	278.61	114.68
	Model 4	143.62	235.44	**102.13**	115.59	237.14	**81.13**
			***n = 200 + 200***			***n = 200 + 200***	
	Model 1	**126.56**	252.88	251.70	**192.47**	244.17	225.63
$\underset{gu^{\prime }}{\sum }E_{gu^{\prime }}$	Model 2	262.92	371.69	**230.25**	297.68	348.46	**215.70**
	Model 3	**139.27**	324.55	215.12	**141.28**	328.64	191.61
	Model 4	189.69	287.39	**169.86**	164.13	280.21	**149.95**

Sum of mean absolute differences between theoretic and estimated penetrance function for 100 case-control data sets in the low and high risk scenario for different sample sizes. Bold numbers mark the best model fit comparing neural networks and logistic regression models. DV = design variables.

With decreasing sample sizes, the model fit again becomes worse and the variance increases (data not shown). If the sample size is 500 + 500 subjects, neural networks again have the best model fit for the first three risk models in the high risk scenario. In the low risk scenario, this is only true for the first and the third risk model. A sample size of just 200 + 200 subjects leads to a considerably worse model fit of neural networks. In this situation, logistic regression models with design variables coding the genotype have the best model fit for the second and fourth risk model in both risk scenarios. Neural networks still have the best model fit if the gene has a masking effect only.

#### Real data application

**Figure 7:**
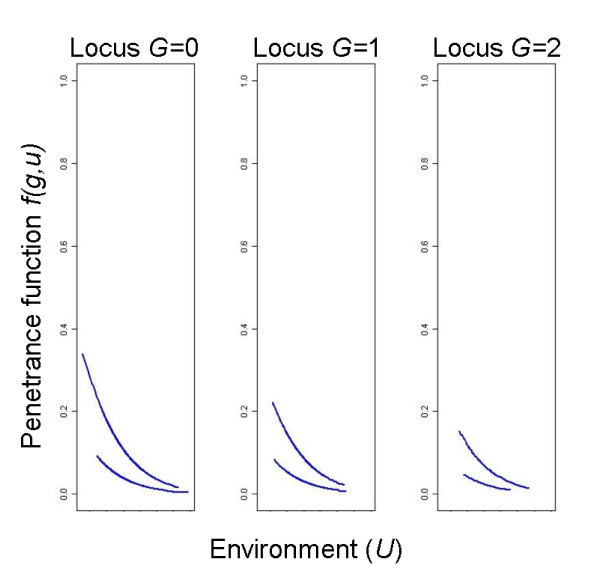
**Real data set application.** Prediction of the neural network using the whole data set. Two lines per genotype result from the inclusion of a binary confounding factor in the analysis. 138 cases and 1599 controls

**Figure 8:**
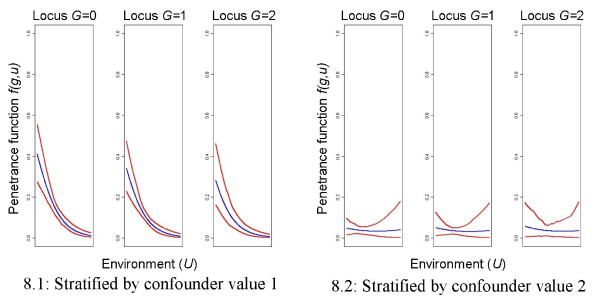
**Real data set application, stratified analysis.** Mean predictions of the neural network over 100 bootstrap replications (blue lines) and 95% bootstrap confidence intervals (red lines). *n *= 112 + 916 (cases+controls) for value 1 of the confounding factor and *n *= 26 + 683 (cases+controls) for value 2 of the confounding factor.

The results for the real data application are shown in Figure 7 (whole data set) and Figure 8 (stratified analysis). A neural network without any hidden neuron is chosen as best neural network. It shows that the environmental factor in general has a decreasing effect on the disease risk and that the number of mutated alleles defines the slope of this risk decrease. If one or two mutated alleles are present, the risk is lower and the risk decrease is weaker as for the homozygous wildtype genotype. We also see a strong influence of the included binary confounding factor.

## Discussion

In this paper, we studied the ability of neural networks and logistic regression models to capture different types of gene-environment interactions. Neural networks were able to predict the theoretic risk models in all sixteen investigated situations such that the prediction intervals contained the true underlying risk models in most situations and were thus superior to logistic regression models. Logistic regression models without design variables completely failed to model the constant effects. Employing design variables led to a considerably better model fit only when average values over the 100 data sets were considered. Single predictions for one data set often had a misleading form and did not distinguish between linear and non-linear components especially for the first two risk models. Nevertheless for risk model 4, logistic regression models using design variables provided the best model fit compared with neural networks as could be seen by the mean absolute differences although the prediction interval did not include the whole true risk model. However, the reasoning behind this fact is still unknown. The real data set application showed the general usability of neural networks in real life situations. Neural networks discovered different risk slopes for each genotype, which also became obvious from the corresponding bootstrap confidence intervals.

Neural networks do not use interaction terms. In our application, they mainly needed one or two hidden neurons if the environmental factor had an effect (risk models by [14]) and they needed one hidden neuron if the locus only had a masking effect and two hidden neurons if the locus had an own main effect (risk models representing a masking effect of the genetic factor). For logistic regression, the correct main effect models were mainly selected for the genetic and the environmental model as best models based on BIC and full models were selected for the additive and interaction model. Thus, the latter two risk models cannot be distinguished from each other based on the co-variables included. Logistic regression models mainly needed an interaction term to model the underlying risk models representing a masking effect of the genetic factor regardless of whether the genotype was coded co-dominant or using design variables (data not shown).

Logistic regression models belong to the class of generalized linear models and as such are limited in their modeling capacity to linearly separable data. On the contrary, neural networks can adapt to any piecewise continuous function. Since linear and non-linear relationships can be modeled simultaneously, neural networks are a promising tool if little is known about the exact relationship between co-variables and a response variable or especially, if a non-linear relationship is assumed.

**Figure 9:**
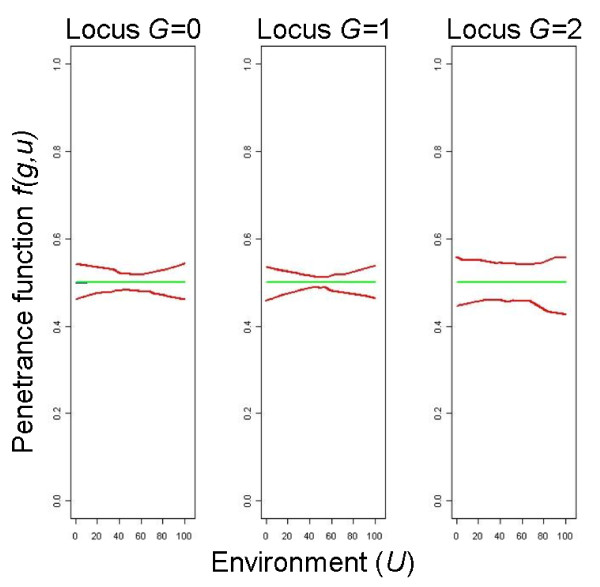
**Mean prediction of the neural network.** Risk model assumes no association. Mean prediction of the neural network $\frac{1}{100}\overset{100}{\underset{k=1}{\sum }}{\hat{f}}^{\left(k\right)}(g,u^{\prime })$ for all *u*^*′ *^= 0, 0.1, 0.2,…,100 and *g *= 0,1,2. Green lines refer to the theoretic risk model, blue lines to the mean predictions, and red lines to the pointwise prediction intervals. *n *= 1,000 + 1,000.

In addition, we showed for simulated data assuming neither an association of the genetic nor an association of the environmental factor that neural networks also have a good model fit in this situation (see Figure 9 for sample size *n *= 1,000 + 1,000). Neural networks without any hidden layer were selected for all but two data sets, thus, being equivalent to logistic regression models including both main effects. For only two data sets with sample size *n *= 200 + 200, a neural network with one hidden neuron was selected.

Thus, our results suggest that neural networks can be a valuable approach already in the situation of 500 cases and 500 controls. However, there are two main drawbacks of neural networks. First, the computing time needed to train them is very high. In our application, the analyses for one situation (100 replications, six network topologies each) sometimes took more than 30 hours. Second, neural networks are still considered as black-box approach since both network topology and trained weights have no direct interpretation. Thus, there is no established way for model selection and parameter testing. One possibility to estimate the effect of a co-variable is provided by the concept of generalized weights [20]. The aim of this paper was to investigate the general modeling ability of neural networks as a first step. Further research should to be devoted to the missing interpretability of trained neural networks.

We assumed the environmental factor to be uniformly distributed over the interval [0,100]. In practice, bell-shaped distributions for environmental factors might be also of interest. Here, it can be expected that a higher sample size is necessary to enable the statistical method to detect the true shape of the underlying risk function also at the margins. Additionally, we assumed the minor allele frequency to be 30%. In a sensitivity analysis, we repeated the simulation study with a minor allele frequency of 5% (see Table 4). Neural networks again outperformed logistic regression models using the risk models by Amato et al. [14]. Using the risk models representing a masking effect of the genetic factor, both, logistic regression models as well as neural networks had problems to fit the data. Due to very small cell frequencies or even empty cells, this was especially true for risk models 3 and 4 where the non-mutated allele masks the effect of the environmental factor. Here, the prediction intervals of neural networks did not even include the true risk model in any situation. Null models and main effect models only including the genetic factor were often used for logistic regression models neglecting the effect of the environmental factor. For neural networks, topologies without hidden neuron were mainly selected.

**Table 4:** Differences between theoretic and estimated penetrance functions (sensitivity analysis: low minor allele frequency)

			**High risk scenario**			**Low risk scenario**	
		**Neural network**	**Logistic regression**	**Logistic regression (DV)**	**Neural network**	**Logistic regression**	**Logistic regression (DV)**
			***n = 1000 + 1000***			***n = 1000 + 1000***	
	Genetic model	**80.29**	80.39	303.07^∗^	**87.65**	209.74	249.96
$\underset{gu^{\prime }}{\sum }E_{gu^{\prime }}$	Environmental model	**79.60**	278.32	277.18	**78.18**	170.94	170.94
	Additive model	**74.67**	369.57	443.10	**92.18**	303.98	348.50
	Interaction model	**180.02**	415.60	541.02^∗^	**191.77**	327.44	481.62^∗^
	Model 1	**113.62**	244.87	375.43^∗^	**179.23**	226.03	355.59^∗^
$\underset{gu^{\prime }}{\sum }E_{gu^{\prime }}$	Model 2	**232.75**	389.70	472.47^∗^	**318.57**	346.57	460.08^∗^
	Model 3	253.00	**230.12**	232.20	256.38	**253.67**	254.80
	Model 4	133.91	126.27	**97.92**	138.28	132.11	**93.04**

Sum of mean absolute differences between theoretic and estimated penetrance function for 100 case-control data sets in the low and high risk scenario for different sample sizes. Bold numbers mark the best model fit comparing neural networks and logistic regression models. DV = design variables. ^∗^Predictions were calculated for all models that do not have unspecified parameters due to empty cells.

## Conclusions

To the best of our knowledge, neural networks have not been used for modeling gene-environment interactions so far. In other contexts, MLPs were clearly superior to logistic regression models [21,22]. Previously, we have successfully employed neural networks for the analysis of gene-gene interactions in simulation studies [10]. This paper shows that the advantages of neural networks are even more pronounced when modeling gene-environment interactions with continuous environmental factors.

In practice, neural networks can be applied in case-control studies to investigate the common effect of two genetic factors or one genetic and one environmental factor. Since the functional form of the model has not to be specified in neural networks, it has neither to be known whether the two involved factors indeed have an effect on the disease nor whether an interaction between both factors is present. The prediction of a neural network generates insight in the kind of relationship between co-variables and disease, for example, whether the underlying relationship is non-linear or whether there are different relationships per genotype. Thus, although there is still need for further research regarding the interpretability of neural networks, neural networks are already a valuable statistical tool especially for exploratory analyses and/or when little is known about the functional relationship of risk factors and investigated disease.

## Appendix

### Artificial neural networks

The general idea of a multilayer perceptron (MLP) is to approximate functional relationships between co-variables and response variable(s). It consists of neurons and synapses that are organized as a weighted directed graph. The neurons are arranged in layers and subsequent layers are usually fully connected by synapses. Each synapse is attached by a weight indicating the effect of this synapse. A positive weight indicates an amplifying, a negative weight a repressing effect. Neural networks have to be trained using a learning algorithm to adjust the synaptic weights according to given data. The learning algorithm minimizes the deviation of predicted output and given response variable measured by an error function.

**Figure 10:**
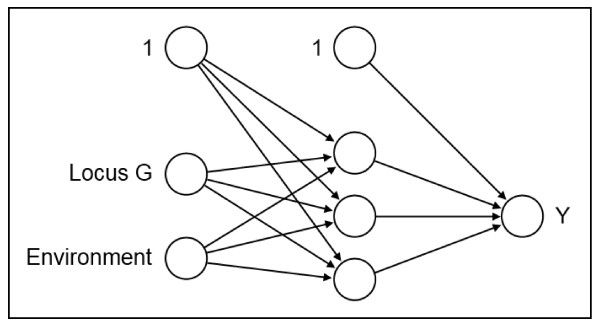
**A multilayer perceptron.** An MLP with one hidden layer consisting of three hidden neurons.

Data passes the MLP as signals. This process starts at the input layer consisting of all co-variables and a constant neuron and it stops at the output layer consisting of the response variable(s). Hidden neurons can be included between the input and output layer in several layers to increase the modeling flexibility. These hidden layers are not directly observable and cannot be controlled by data. See Figure 10 for an MLP with one hidden layer consisting of three hidden neurons that models the functional relationship between the locus *G* and the environmental factor *U* as co-variables and the case-control status *Y * as response variable.

An MLP with one hidden layer is able to fit any piecewise continuous function [23]. Thus, we consider MLPs with at most one hidden layer in this paper. An MLP consisting of *n* + 1 input neurons, *m* hidden neurons, and one output neuron computes the following predicted output

$$\mu (x)=\sigma \left(w_{0}+\overset{m}{\underset{j=1}{\sum }}w_{j}\cdot \sigma \left(\overset{n}{\underset{i=0}{\sum }}w_{\mathrm{ij}}x_{i}\right)\right),$$

where *w*_0_, *w*_*j*_, and *w*_*ij*_, *i *= 0,…,*n*, *j *= 1,…,*m*, denote the weights including intercepts, ***x = (x***_0_*x*_1_,…,*x*_*n*_)^*T*^ the vector of all co-variables including a constant neuron *x*_0_ and *σ* the activation function that maps the output of each neuron to a given range. MLPs are a direct extension of generalized linear models (GLM, [24]) and an MLP without hidden layer is algebraically equivalent to a generalized linear model with *σ* as inverse link function. In this case, trained weights and estimated regression coefficients coincide.

To train neural networks according to the case-control data sets, resilient backpropagation [25] as learning algorithm with cross entropy as error function and logistic function as activation function is used.

## Competing interests

The authors declare that they have no competing interests.

## Author’s contributions

FG planned and carried out the simulation study and drafted the manuscript. IP drafted the manuscript. KB planned the simulation study and drafted the manuscript. All authors read and approved the final manuscript.

## References

[B1] Wray N Goddard M Visscher P Prediction of individual genetic risk of complex disease Curr Opin Genet Dev 2008 18 257 263 10.1016/j.gde.2008.07.006 18682292

[B2] Gibson G Decanalization and the origin of complex disease Nat Rev Genet 2009 10 2 134 140 19119265 10.1038/nrg2502

[B3] Galvan A Ioannidis J Dragani T Beyond genome-wide association studies: genetic heterogeneity and individual predisposition to cancer Trends Genet 2010 26 3 132 141 10.1016/j.tig.2009.12.008 20106545 PMC2826571

[B4] Abazyan B Nomura J Kannan G Ishizuka K Tamashiro K Nucifora F Pogorelov V Ladenheim B Yang C Krasnova I Cadet J Pardo C Mori S Kamiya A Vogel M Sawa A Ross C Pletnikov M Prenatal interaction of mutant DISC1 and immune activation produces adult psychopathology Biol Psychiatry 2010 68 1172 1181 10.1016/j.biopsych.2010.09.022 21130225 PMC3026608

[B5] Hutter C Slattery M Duggan D Muehling J Curtin K Hsu L Beresford S Rajkovic A Sarto G Marshall J Hammad N Wallace R Makar K Prentice R Caan B Potter J Peters U Characterization of the association between 8q24 and colon cancer: gene-environment exploration and meta-analysis BMC Cancer 2010 10 670 10.1186/1471-2407-10-670 21129217 PMC3017062

[B6] Kazma R Babron M Génin E Genetic association and gene-environment interaction: a new method for overcoming the lack of exposure information in controls Am J Epidemiol 2011 173 2 225 235 10.1093/aje/kwq352 21084555

[B7] Docherty S Kovas Y Plomin R Gene-environment interaction in the etiology of mathematical ability using SNP sets Behav Genet 2011 41 141 154 10.1007/s10519-010-9405-6 20978832 PMC3029801

[B8] Tolonen S Laaksonen M Mikkilä V Sievänen H Mononen N Räsänen L Viikari J Raitakari O Kähönen M Lehtimäki T Cardiovascular Risk in Young Finns Study Group: Lactase gene C/T13910 polymorphism, calcium intake, and pQCT bone traits in finnish adults Calcified Tissue Int 2011 58 153 161 10.1007/s00223-010-9440-6 21136048

[B9] Bammann K Pohlabeln H Pigeot I Jöckel K Use of an artificial neural network in exploring the dose-response relationship between cigarette smoking and lung cancer risk in male Far East J Theor Stat 2005 16 2 285 302

[B10] Günther F Wawro N Bammann K Neural networks for modeling gene-gene interactions in association studies BMC Genet 2009 10 87 20030838 10.1186/1471-2156-10-87 PMC2817696

[B11] Gago J Landín M Gallego P Artificial neural networks modeling the in vitro rhizogenesis and acclimatization of Vitis vinifera L J Plant Physiol 2010 167 1226 1231 10.1016/j.jplph.2010.04.008 20542352

[B12] Lin RH Chuang CL A hybrid diagnosis model for determining the types of the liver disease Comput Biol Med 2010 40 7 665 670 10.1016/j.compbiomed.2010.06.002 20591425

[B13] Ioannidis J Trikalinos T Law M Carr A HIV Lipodystrophy Case Definition Study Group: HIV lipodystrophy case definition using artificial neural network modelling Antivir Ther 2003 8 435 441 14640391

[B14] Amato R Pinelli M D’Andrea D Miele G Nicodemi M Raiconi G Cocozza S A novel approach to simulate gene-environment interactions in complex diseases BMC Bioinf 2010 11 8 10.1186/1471-2105-11-8 PMC2824681 20051127

[B15] Bishop C Neural Networks for Pattern Recognition 1995 New York: Oxford University Press

[B16] Schwarz G Estimating the dimension of a model Ann Stat 1978 6 461 464 10.1214/aos/1176344136

[B17] Development Core Team R R: A Language and Environment for Statistical Computing. Vienna 2009 Austria: R Foundation for Statistical Computing [http://www.R-project.org]. [ISBN 3-900051-07-0]

[B18] Günther F Fritsch S neuralnet: Training of neural networks R J 2010 2 30 38

[B19] Efron B Tibshirani R An Introduction to the Bootstrap 1993 Boca Raton: Chapman and Hall

[B20] Intrator O Intrator N Interpreting neural-network results: a simulation study Comput Stat Data An 2001 37 373 393 10.1016/S0167-9473(01)00016-0

[B21] Savegnago R Nunes B Caetano S Ferraudo A Schmidt G Ledur M Munari D Comparison of logistic and neural network models to fit to the egg production curve of White Leghorn hens Poult Sci 2011 90 3 705 711 10.3382/ps.2010-00723 21325246

[B22] Liew P Lee Y Lin Y Lee T Lee W Wang W Chien C Comparison of artificial neural networks with logistic regression in prediction of gallbladder disease among obese patients Digest Liver Dis 2007 39 4 356 362 10.1016/j.dld.2007.01.003 17317348

[B23] Hecht-Nielsen R Neurocomputing 1990 Reading: Addison-Wesley

[B24] McCullagh P Nelder J Generalized Linear Models 1983 London: Chapman and Hall

[B25] Riedmiller M Advanced supervised learning in multi-layer perceptrons – from backpropagation to adaptive learning algorithms Int J Comput Stand Interf 1994 16 265 275 10.1016/0920-5489(94)90017-5

